# hnRNP A1 and hnRNP C associate with *miR‐17* and *miR‐18* in thyroid cancer cells

**DOI:** 10.1002/2211-5463.13409

**Published:** 2022-04-24

**Authors:** Maria Gabriela Pereira dos Santos, Guilherme Henrique Gatti da Silva, Helder Yudi Nagasse, Cesar Seigi Fuziwara, Edna T. Kimura, Patricia Pereira Coltri

**Affiliations:** ^1^ Departamento de Biologia Celular e do Desenvolvimento Instituto de Ciências Biomédicas Universidade de São Paulo Brazil; ^2^ Present address: National Center for Tumor Diseases (NCT) Dresden Fetscherstraße 74 Dresden 01307 Germany

**Keywords:** hnRNP A1, hnRNP C, *miR‐17‐92* cluster, miRNA, pre‐mRNA splicing, thyroid

## Abstract

Heterogeneous nuclear ribonucleoproteins (hnRNPs) are essential players in the regulation of gene expression. The majority of the twenty different hnRNP proteins act through the modulation of pre‐mRNA splicing. Most have been shown to regulate the expression of critical genes for the progression of tumorigenic processes and were also observed to be overexpressed in several types of cancer. Moreover, these proteins were described as essential components for the maturation of some microRNAs (miRNAs). In the human genome, over 70% of miRNAs are transcribed from introns; therefore, we hypothesized that regulatory proteins involved with splicing could be important for their maturation. Increased expression of the *miR‐17‐92* cluster has already been shown to be related to the development of many cancers, such as thyroid, lung, and lymphoma. In this article, we show that overexpression of hnRNP A1 and hnRNP C in BCPAP thyroid cancer cells directly affects the expression of *miR‐17‐92* miRNAs. Both proteins associate with the 5′‐end of this cluster, strongly precipitate miRNAs *miR‐17* and *miR‐18a* and upregulate the expression of *miR‐92a*. Upon overexpression of these hnRNPs, BCPAP cells also show increased proliferation, migration, and invasion rates, suggesting upregulation of these proteins and miRNAs is related to an enhanced tumorigenic phenotype.

## Graphical abstract

hnRNP A1 and hnRNP C proteins associate with miRNAs *miR‐17* and *miR‐18* in thyroid cancer cells. Overexpression of these proteins leads to increased cell growth and invasion in thyroid cancer cells, enhancing tumorigenic characteristics.

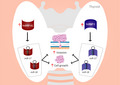

**Abbreviations**
EMT: epithelial‐mesenchymal transition
G418: geneticin
hnRNPs: heterogeneous nuclear ribonucleoproteins
miRNAs: microRNAs
PTEN: phosphatase and tensin homolog

Cancer results from several alterations in gene expression that can occur at transcriptional, post‐transcriptional, and translational levels. Such alterations cause cells to acquire a set of characteristics different from normal cells but familiar to most cancer cells [1]. These characteristics derive from several mechanisms that lead cells, for instance, to proliferate uncontrollably and migrate to different tissues, allowing them to adapt to other conditions and microenvironments [2, 3]. In addition, many microRNAs (miRNAs) have been associated with alterations in gene expression and cancer development. miRNAs are non‐coding molecules, usually 18–22 nucleotides in length, that can act from chromatin modeling to translational repression, affecting cell metabolism, proliferation and differentiation [4, 5, 6, 7]. These processes are essential during cancer progression, and many studies have already demonstrated that miRNAs are key pieces in tumorigenesis [8].

MiRNAs are entirely or partially complementary to the 5′ or 3′ UTR of target mRNAs. By binding to these sequences, miRNAs can regulate chromatin remodeling, translation, and RNA degradation, therefore affecting gene expression of possible oncogenes or tumor suppressors [9]. The expression of a combination of miRNAs is important for determining the function of a specific cell [10]. Several alterations in the expression of miRNAs have been reported in leukemia, lung, pancreas, stomach, and ovary cancers [6, 11, 12]. The miRNA cluster *miR‐17‐92* is transcribed from an intron of the *MIR17HG* gene (chromosome 13) and consists of 7 miRNAs, *miR‐17‐5p*, *miR‐17‐3p*, *miR‐18a*, *miR‐19a*, *miR‐20a*, *miR‐19b,* and *miR‐92a* [13, 14, 15]. The aberrant expression of these miRNAs is related to different diseases, such as thyroid cancer, chronic lymphocytic leukemia, chronic myeloid leukemia, lung cancer, lymphomas, and cystic fibrosis [15, 16, 17, 18, 19]. Considering the intronic location of this cluster, we hypothesized pre‐mRNA splicing regulatory proteins would be important for its expression. We have previously identified hnRNP A1 and hnRNP C proteins enriched in spliceosomes assembled on introns containing *miR‐18a* and *miR‐19a* [20], raising the hypothesis that these proteins could be associated with biogenesis of this miRNA cluster.

Several hnRNP proteins could affect the splicing regulation of the intron containing *miR‐17‐92* cluster. Many hnRNPs are directly related to the generation of isoforms with oncogenic characteristics, affecting the cell phenotype [21]. hnRNPs have RNA‐binding capacity and form a large family of proteins associated with RNA polymerase II transcripts [22]. Both hnRNP A1 and hnRNP C are important regulators of c‐MYC expression, a critical transcription factor for the progression of many tumors [23, 24, 25]. Moreover, it has been shown these proteins often recognize splicing regulatory elements that are present in pre‐mRNAs, especially the intronic splicing enhancers, and intronic splicing silencers, leading to different alternative splicing patterns. Thus, altered levels of these proteins might regulate the alternative splicing patterns observed in the cells controlling abundance of pro‐ or anti‐apoptotic isoforms, or regulating cell cycle, for example [23, 26, 27]. hnRNP expression levels are altered in several types of cancer. hnRNP A1 is overexpressed in many tumors, such as Burkitt's lymphoma, leukemia, multiple myeloma, prostate, and lung cancer [28, 29, 30]. hnRNP C has been associated with metastatic processes in Glioblastoma multiforme cells by reducing the levels of PDCD4 protein, an important tumor suppressor [31].

hnRNP proteins are also required for the maturation of some miRNAs [32]. Previous studies have shown hnRNP A1 is associated with *miR‐18a*, and this interaction is important for its maturation [33, 34, 35]. We hypothesized hnRNP A1 and hnRNP C could bind to the intron and regulate the biogenesis of *miR‐17‐92* miRNA cluster, therefore promoting increased expression of these miRNAs and leading to altered cell phenotype. In this work, we observed both hnRNP A1 and hnRNP C stimulate the biogenesis of *miR‐17‐92* components, strongly associating with miRNAs *miR‐17* and *miR‐18a*, transcribed from the 5′‐end of the cluster. Increased expression of these proteins and miRNAs resulted in enhanced cell proliferation and migration phenotypes in thyroid cancer cells.

## Materials and methods

### Cell culture and transfection

BCPAP cells are derived from pappilary thyroid cancer and were kindly provided by M. Santoro (University Federico II of Naples, Italy). These cells were cultivated in Dulbecco's modified Eagle's medium (Thermo, Waltham, MA, USA) supplemented with 10% FBS, 100 U·mL^−1^ penicillin, 1 µg·mL^−1^ streptomycin and 100 µg·mL^−1^ amphotericin at 37 °C and 5% CO_2_ atmosphere. Plasmids pFLAG‐hnRNP A1 and pFLAG‐hnRNP C were transfected into BCPAP cells using Lipofectamine 2000 (Invitrogen, Carlsbad, CA, USA), according to the manufacturer instructions. Empty pFLAG was transfected in the same cells as a control. Cells were selected by gradually increasing the concentration of geneticin (G418) up to 1000 µg·mL^−1^, generating stably transfected cells.

### Immunoprecipitation

BCPAP cells overexpressing hnRNP A1 and hnRNP C and control cells expressing only the FLAG epitope were cultured for 48 h in 100 mm Petri dishes. After cells were collected, extracts were prepared in buffer A (10 mm KCl, 1.5 mm MgCl_2_, 20 mm Tris [pH 7.5], 0.5 mm DTT), and then they were homogenized using Douncer (Wheaton, NJ, USA). Under rotary agitation, these extracts were immunoprecipitated by incubation with protein A‐sepharose coupled to anti‐FLAG M2 (Sigma, San Luis, MI, USA) for 16 h at 4 °C, under rotary agitation. After incubation, the resin was washed three times using buffer PBS 1X, to remove the non‐specific binding, and proteins were eluted using 25 µg·µL^−1^ FLAG peptide for 2 h. Elution fractions were used to perform RNA extraction and western blot.

### RNA extraction and quantitative real‐time PCR

Total RNA was extracted from indicated cells using Trizol Reagent (Invitrogen, Waltham, MA, USA) and precipitated using sodium acetate and ethanol. cDNAs were prepared immediately after extraction using Superscript IV RT enzyme (Life Technologies, Carlsbad, CA, USA) and random primers. Quantitative real‐time PCR (qPCR) was performed with the SYBR Green reagent (Thermo Fisher Scientific, Waltham, MA, USA) and specific primers for hnRNP A1 and hnRNP C mRNAs, pre‐*miR‐17*, pre‐*miR‐18a*, pre‐*miR19a*, pre‐*miR‐92a*, RNU6B and β‐actin. Relative gene expression was calculated by the $2^{‐\Delta \Delta C_{t}}$ method [36]. Fold changes in gene expression were normalized by amplification with RNU6B primers. Statistical analysis was performed as described below.

### Cell growth and antimiR‐19a transfection

BCPAP‐hnRNP A1, BCPAP‐hnRNP C, and BCPAP‐FLAG cells were seeded (2.5 × 10^4^) in 6‐well plates (0 h) and cultured for up to 24 h. After seeding (time 0) and after 6 and 24 h of growth, cells were trypsinized, collected, stained with 0.4% trypan blue solution (Sigma) in 1 : 1 ratio, and viable cells were counted using Countess II FL (Life Technologies). Each assay was performed in triplicate and using three different biological samples.

5 × 10^4^ BCPAP‐hnRNP A1, BCPAP‐hnRNP C, and BCPAP‐FLAG cells were seeded in 6‐well plates and incubated for 24 h. After that, these cells were transfected with 25 nm of antimiR‐19a (miRVana, Thermo catalogue number 4464084) and the negative control (mirVana, Thermo catalogue number 4464076) using Lipofectamine 2000 (Life Technologies). Cell growth after 24 and 72 h post‐transfection was evaluated using trypan blue.

### Migration and invasion assays

Migration and invasion assays were performed using chambers with a transwell membrane (pore size of 8.0 μm; Corning Inc, New York, NY, USA). These assays allowed us to check whether the hnRNP A1 and hnRNP C overexpression affected the migratory and invasive capacity of cells overexpressing these proteins. For migration assays, membranes were uncoated, incubated with PBS (3.2 mm Na_2_HPO_4_, 0.5 mm KH_2_PO_4_, 1.3 mm KCl, 135 mm NaCl, pH 7.4) for 1 h at 37 °C, 5% CO_2_ atmosphere. For invasion assays, membranes were coated with 25 μg Matrigel® (BD Biosciences, East Rutherford, NJ, USA) and incubated for 1 h at 37 °C, 5% CO_2_ atmosphere. 2.5 × 10^4^ cells were suspended in a culture medium containing 1% FBS and plated in the upper chamber, whereas the lower chamber contained a culture medium with 10% FBS. After 8 h at 37 °C, 5% CO_2_, non‐migrating cells on the top chamber were removed using a cotton swab, and cells that migrated through the membrane were fixed with 4% paraformaldehyde (PFA) in PBS and stained with 0.5% Crystal Violet. Cells were photographed using a Nikon Eclipse E600 microscope (Nikon, Tokyo, Japan) equipped with optical camera CF160 epifluorescence, and 10 representative fields were counted. Scale bars in the images represent 50 µm.

### Statistical analysis

Results are expressed as mean ± standard deviation (SD). All experimental data were collected from at least three independent experiments to exclude any possible variation caused by differences among cell cultures. Group comparisons were performed using two‐way ANOVA with post‐test Tukey analysis and Student's *t*‐test. *P* < 0.05 was considered to be statistically significant.

### *In* *silico* analysis

*In silico* analysis of hnRNP A1 and hnRNP C expression levels was performed using data with cutoffs above 0.5 FPKM (fragments per kilobase) retrieved from the “Expression Atlas and Cancer Cell Line Encyclopedia” (https://www.ebi.ac.uk/gxa/home). Data were plotted onto a heat map graph.

miRDB [37] was used to find the targets for the miRNAs of the *miR‐17‐92* cluster in thyroid cancer cells. As expected, multiple targets were found for each miRNA. Targets with moderate to higher expression were filtered based on the RPKM method (reads per kilobase of transcript, per million mapped reads), for each cell line. Values above 20 were considered high expression; between 5 and 20 were moderate expression; and between 1 and 5 were considered low expression. Results were plotted onto the graphs shown in Fig. S3.

## Results

### Overexpression of hnRNP A1 and hnRNP C increases expression of *miR‐17‐92* miRNAs in BCPAP cells

We previously found hnRNP A1 and hnRNP C proteins enriched in spliceosomes assembled on introns containing *miR‐18a* and *miR‐19a* in thyroid cancer cells [20].

To assess the physiological relevance of hnRNP A1 and hnRNP C expression, we performed an *in silico* analysis on the basal expression of these proteins in a set of thyroid cancer cell lines (Fig. S1). We observed that hnRNP A1 is moderately expressed in most of the analyzed cell lines, including BCPAP. hnRNP C, on the other hand, shows lower expression in the thyroid cell lines. We then hypothesized that modulation of hnRNP A1 and hnRNP C could affect intronic *miR‐17‐92* cluster members, regulating the expression of the individual miRNAs.

**Fig. 1:**
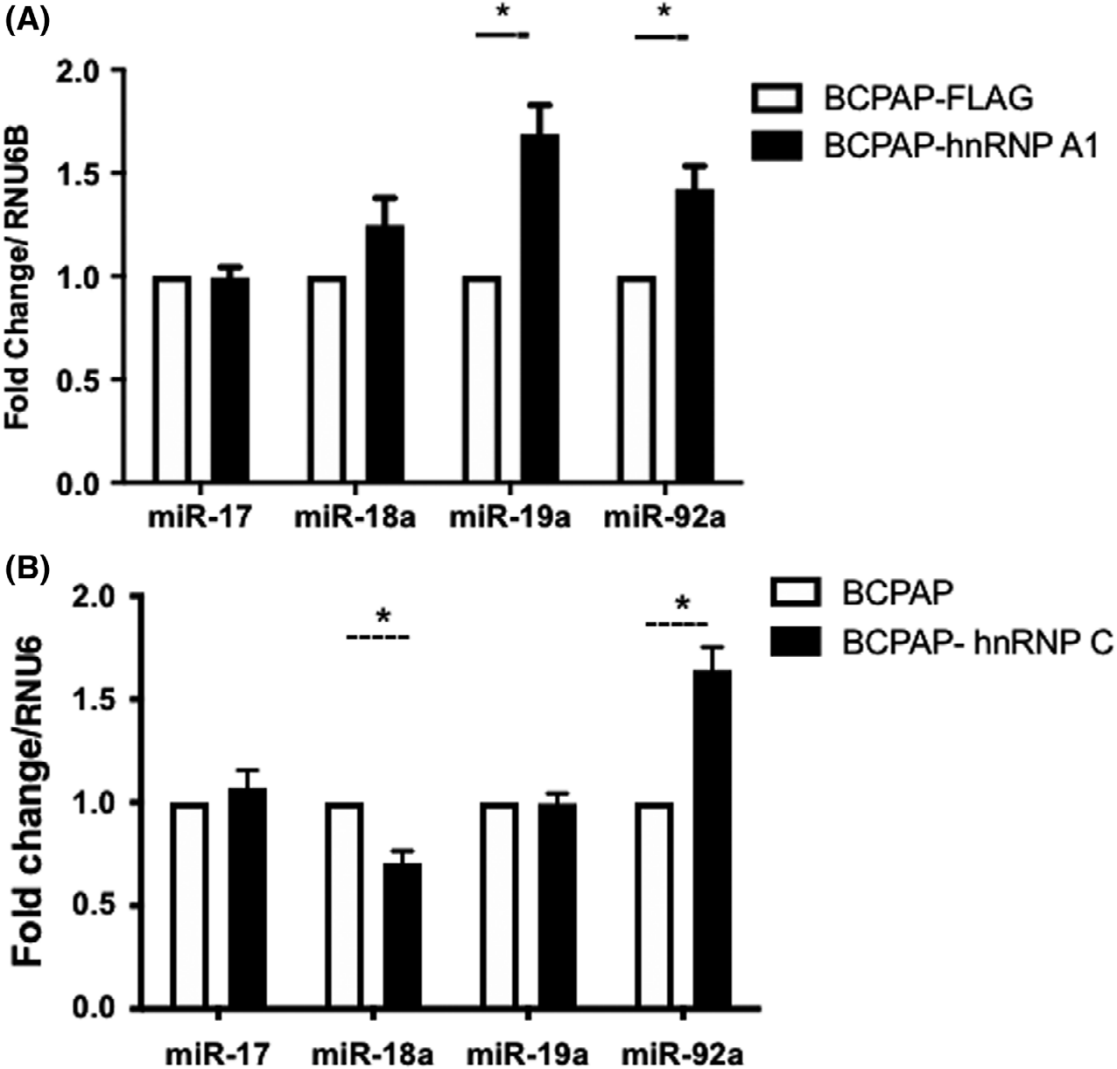
Background miRNA expression. Analysis of *miR‐17*, *miR‐18a*, *miR‐19a,* and *miR‐92a* miRNAs expression in BCPAP cells overexpressing (A) FLAG‐hnRNP A1 and (B) FLAG‐hnRNP C (black bars). The control group expressed only the epitope FLAG (white bars). Normalization was performed upon amplification of the RNU6B gene to calculate fold change. Error bars represent standard deviations calculated from three independent experiments. Group comparisons were performed using two‐way ANOVA with post‐test Tukey analysis and Student's *t*‐test. *P < 0.05.

To investigate this hypothesis, we used BCPAP, a papillary thyroid cancer cell line carrying BRAF^V600E^ mutation [16] to overexpress hnRNP A1 and hnRNP C. The overexpression of both proteins was confirmed after comparison with cells expressing only the FLAG epitope (Fig. S2). Through RT‐qPCR and using specific primers to detect *miR‐17‐92* cluster components (*miR‐17*, *miR‐18a*, *miR‐19a,* and *miR‐92a*), we observed the expression of miRNAs in cells overexpressing hnRNP A1 and hnRNP C compared with control cells (BCPAP‐FLAG) (Fig. 1). Our *miR‐17* primers amplify both *miR‐17‐5p* and *miR‐17‐3p*, and to accomplish that, we will refer to “*miR‐17*” hereafter. Importantly, overexpression of hnRNP A1 in BCPAP cells reflects in a significant increase of *miR‐19a* and *miR‐92a* levels compared with cells expressing only the FLAG epitope. On the other hand, overexpression of hnRNP C leads to a decrease in *miR‐18a* and an increase of *miR‐92a* levels. These results suggest that overexpression of both hnRNP A1 and hnRNP C affect the expression of these miRNAs in thyroid tumor cells.

**Fig. 2:**
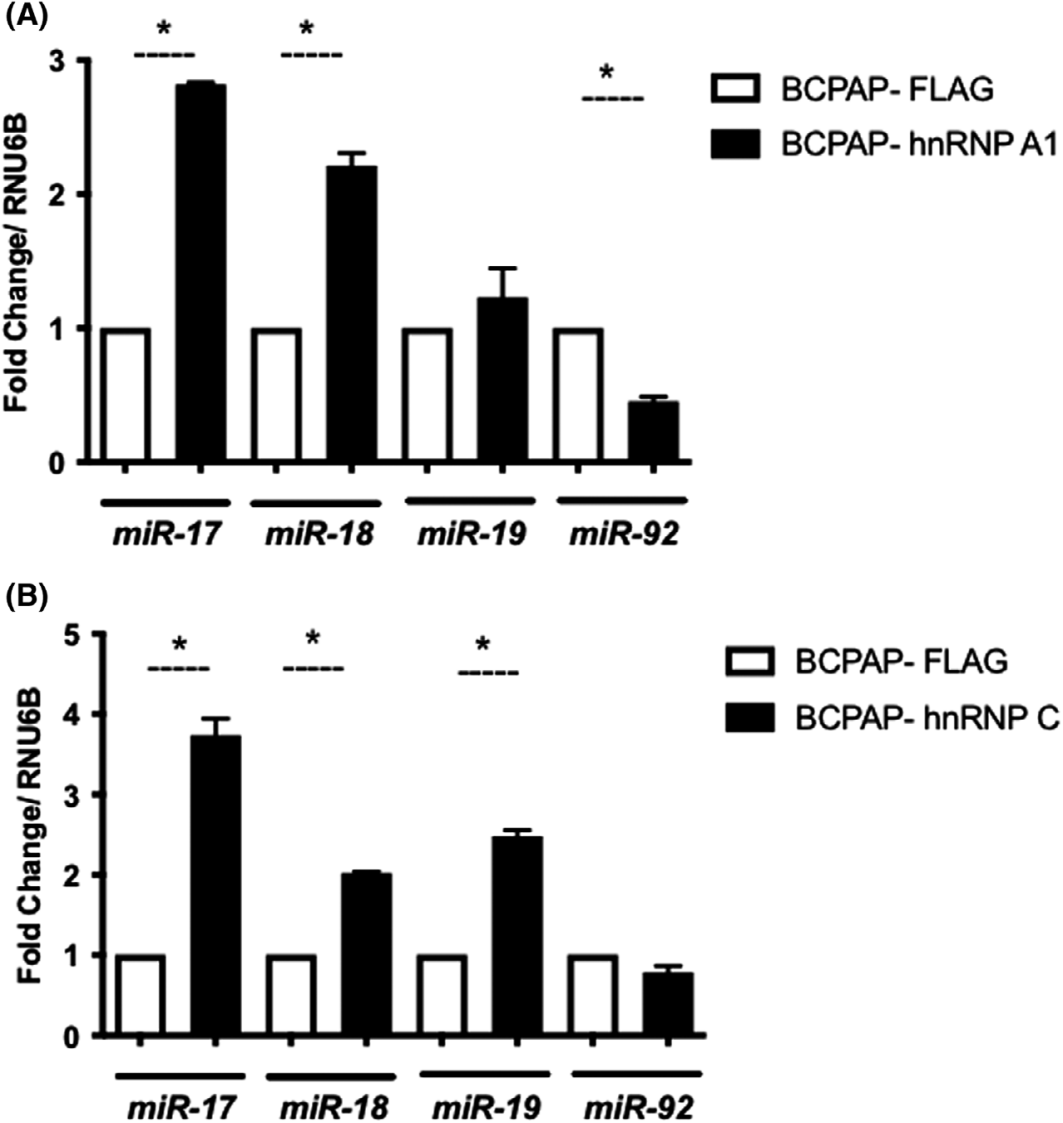
Immunoprecipitation analysis. Immunoprecipitation analysis of (A) FLAG‐hnRNP A1 and (B) FLAG‐hnRNP C (black bars). BCPAP cells transfected with the empty pFLAG vector were used as a control in immunoprecipitations. Elution samples were analyzed by RT‐qPCR using specific primers. Fold change expression was normalized upon the expression of RNU6B. Error bars represent standard deviations calculated from three independent measurements. Group comparisons were performed using two‐way ANOVA with post‐test Tukey analysis and Student's *t*‐test. *P < 0.05.

We then hypothesized hnRNP A1 and hnRNP C could be binding to sequences within the intron containing the cluster, which would partially explain their effect on biogenesis and expression of mature miRNAs. To investigate that, we performed immunoprecipitation using anti‐FLAG antibody in BCPAP‐hnRNP A1 and BCPAP‐hnRNP C whole‐cell extracts. As controls, BCPAP cells expressing only the FLAG epitope were used (Fig. 2). The results revealed hnRNP A1 was significantly associated with the pre‐miRNA regions of *miR‐17* and *miR‐18a*. Importantly, hnRNP A1 did not associate with *miR‐92a*, as shown by this experiment. The observed profile indicates the binding activity of hnRNP A1 decreases along the extension of the cluster, as *miR‐17* and *miR‐18a* were strongly precipitated on this assay. Analysis of possible binding regions for these proteins revealed hnRNP A1 binding site in the 5′ region of the pri‐miRNA, which might explain the higher concentration of the protein associated with miRNAs in the 5′‐end of the cluster. These results support previous studies, which report an important association of hnRNP A1 with *miR‐18a* [33]. This association facilitates Drosha's action through its association with the terminal loop of pri‐miR‐18a leading to processing and maturation of this miRNA [33].

hnRNP C strongly associates with *miR‐17*, *miR‐18a*, and *miR‐19a* (Fig. 2). The levels of *miR‐92* did not change on immunoprecipitated hnRNP C. *In silico* analysis revealed a possible binding site at the 5′ region of the pri‐miRNA, suggesting the protein might also be bound to the 5′‐end of this molecule. This explains the increased precipitation of miRNAs transcribed from the 5′‐end of this cluster and confirms that the protein is bound to this region.

### HnRNP A1 and hnRNP C overexpression are associated with tumorigenic processes

Altered expression levels of some hnRNPs are directly associated with tumorigenic processes [27, 38]. Our results indicated that increased expression of hnRNP A1 and hnRNP C resulted in upregulation of miRNAs of the *miR‐17‐92* cluster. Considering *miR‐17‐92* miRNAs show high expression in more aggressive thyroid cancers, as for example anaplasic thyroid cancer, we investigated the number of target sequences for *miR‐17‐92* in thyroid cell lines [19, 39]. This analysis revealed that genes associated with cell kinetics have more target sequences for *miR‐17‐92* miRNAs. Among these were RAB11, AKT3, and WNT5, which are known to control proliferation, migration, and invasion (Fig. S3) [40, 41, 42]. We thus reasoned that altered expression of miRNAs could directly cause cellular changes that affect thyroid cancer biology [28, 29]. miRNAs from this cluster are involved with extensive pro‐tumoral signaling, for instance, via downregulation of tumor suppressor gene phosphatase and tensin homolog (PTEN) and by regulating E2F family transcription factors [43, 44]. Therefore, we investigated the effects of hnRNP A1 and hnRNP C overexpression and the increased processing of these miRNAs on cells kinetics.

**Fig. 3:**
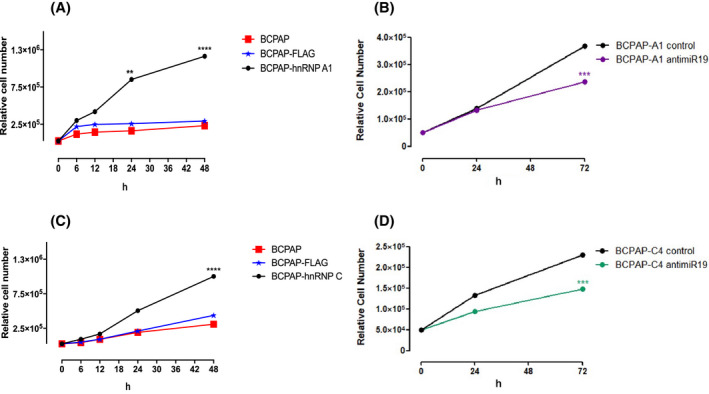
Cell growth of BCPAP cells. Growth of BCPAP cells overexpressing (A, B) hnRNP A1 and (C, D) hnRNP C. (A) BCPAP‐hnRNP A1 growth was evaluated throughout 48 h, red line represents untransfected cells, light blue line represents BCPAP‐FLAG cells and black represents BCPAP‐hnRNP A1. **P < 0.005. ****P < 0.0005. (B) Growth curve of BCPAP‐hnRNP A1 transfected with antimiR‐19a (purple line) and control antimiRNA (black line). Cell counting started 24 h after transfection with antimiR‐19a or control antimiRNA. ***P < 0.0005. (C) BCPAP‐hnRNP C growth was evaluated throughout 48 h, red line represents untransfected cells, light blue line represents BCPAP‐FLAG cells and black represents BCPAP‐hnRNP C. ****P < 0.0005. (D) Growth curve of BCPAP‐hnRNP C transfected with antimiR‐19a (green line) and control antimiRNA (black line). Cell counting started 24 h after transfection with antimiR‐19a or control antimiRNA. ***P < 0.0005. Viable cells were quantified using Countess II FL Automated Cell Counter. The values represent the means of the triplicates of each group. Group comparisons were performed using two‐way ANOVA with post‐test Tukey analysis and Student's *t*‐test.

We first determined the cell viability of BCPAP cells using the Trypan blue exclusion method. Next, BCPAP‐hnRNP A1 and BCPAP‐hnRNP C cells were cultivated for 6 h, and the percentage of viable and dead cells was quantified using Countess II FL Automated Cell Counters (Thermo Fisher, Waltham, MA) (Fig. S4). We did not observe a significative difference in cell viability in all groups tested. We then investigated cell growth rates after incubating cells for up to 72 h. We observed that cells overexpressing hnRNP A1 and hnRNP C grew twice as much as controls after 72 h. As controls, cells expressing only FLAG epitope and untransfected BCPAP cells were used (Fig. 3).

As overexpression of hnRNP A1 and hnRNP C led to increased expression of miRNAs of *miR‐17‐92* cluster, we then used a miRNA inhibitor to suppress the effect of one of the miRNAs of this cluster and analyze cell growth. The transfection of antimiR‐19a (miRVana; Thermo) in hnRNP A1 and hnRNP C overexpressing cells resulted in reduction in 60% of cell growth in comparison with cells transfected with control antimiRNA, after 24 and 72 h (Fig. 3B,D). Importantly, this result indicates the miRNAs from this cluster are modulating cell growth in BCPAP cells.

**Fig. 4:**
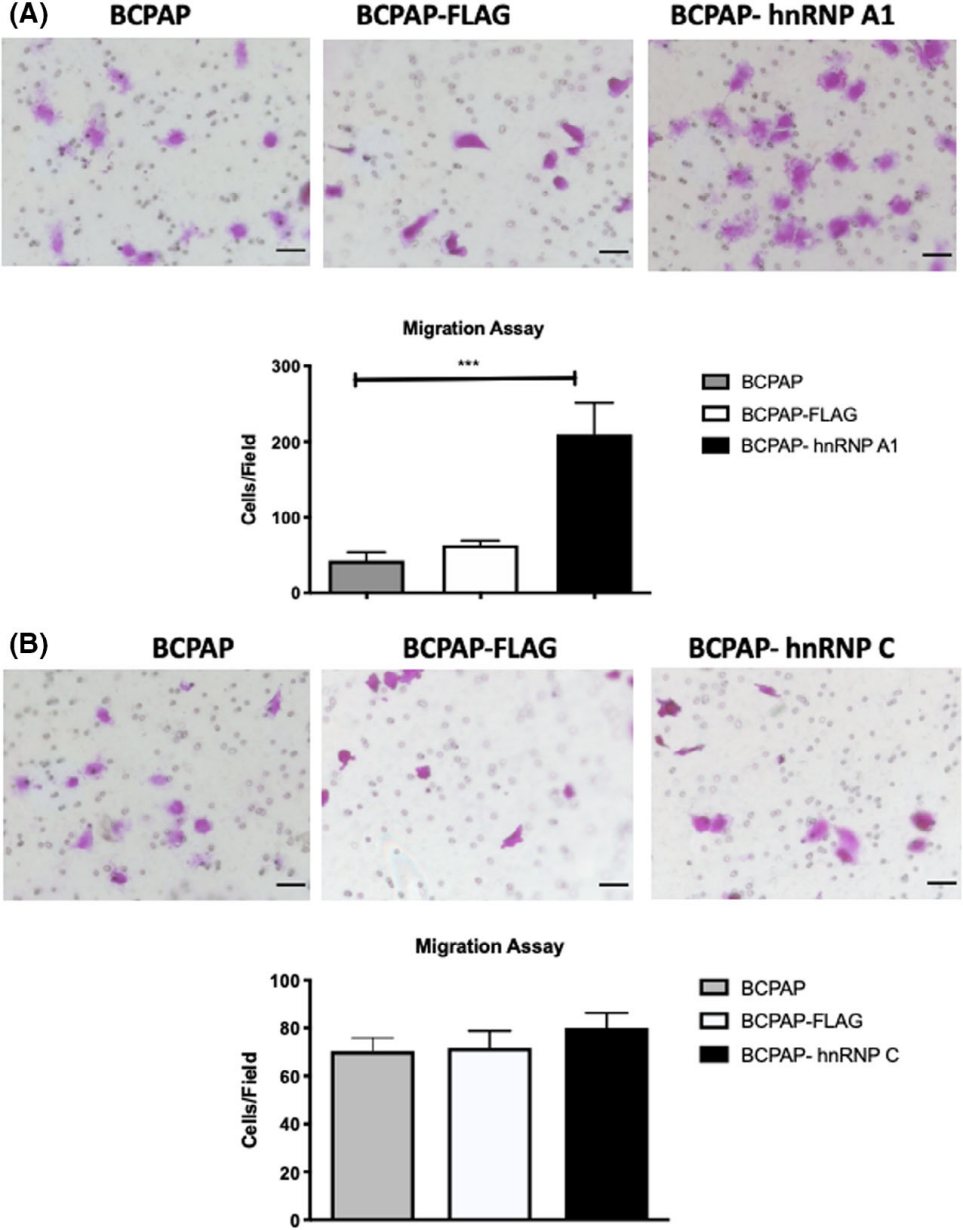
Transwell assays to evaluate cell migration. Assays were performed on (A) BCPAP‐hnRNP A1 and (B) BCPAP‐hnRNP C cells. BCPAP (untransfected) and BCPAP‐FLAG cells were used as controls. Assays were performed for 6 h. Representative images of the cells traversing the transwell are shown for BCPAP, BCPAP‐FLAG and (A) BCPAP‐hnRNP A1 or (B) BCPAP‐hnRNP C, and the quantification of approximately 10 fields is shown on the graph below (gray bars for BCPAP, white bars for BCPAP‐FLAG and black bars for overexpressing cells). The *y*‐axis indicates the average number of cells per field. Error bars represent standard deviations calculated from 10 fields of at least three different biological samples. Group comparisons were performed using two‐way ANOVA with post‐test Tukey analysis and Student's *t*‐test. ***P < 0.0005. Scale bars represent 50 µm.

**Fig. 5:**
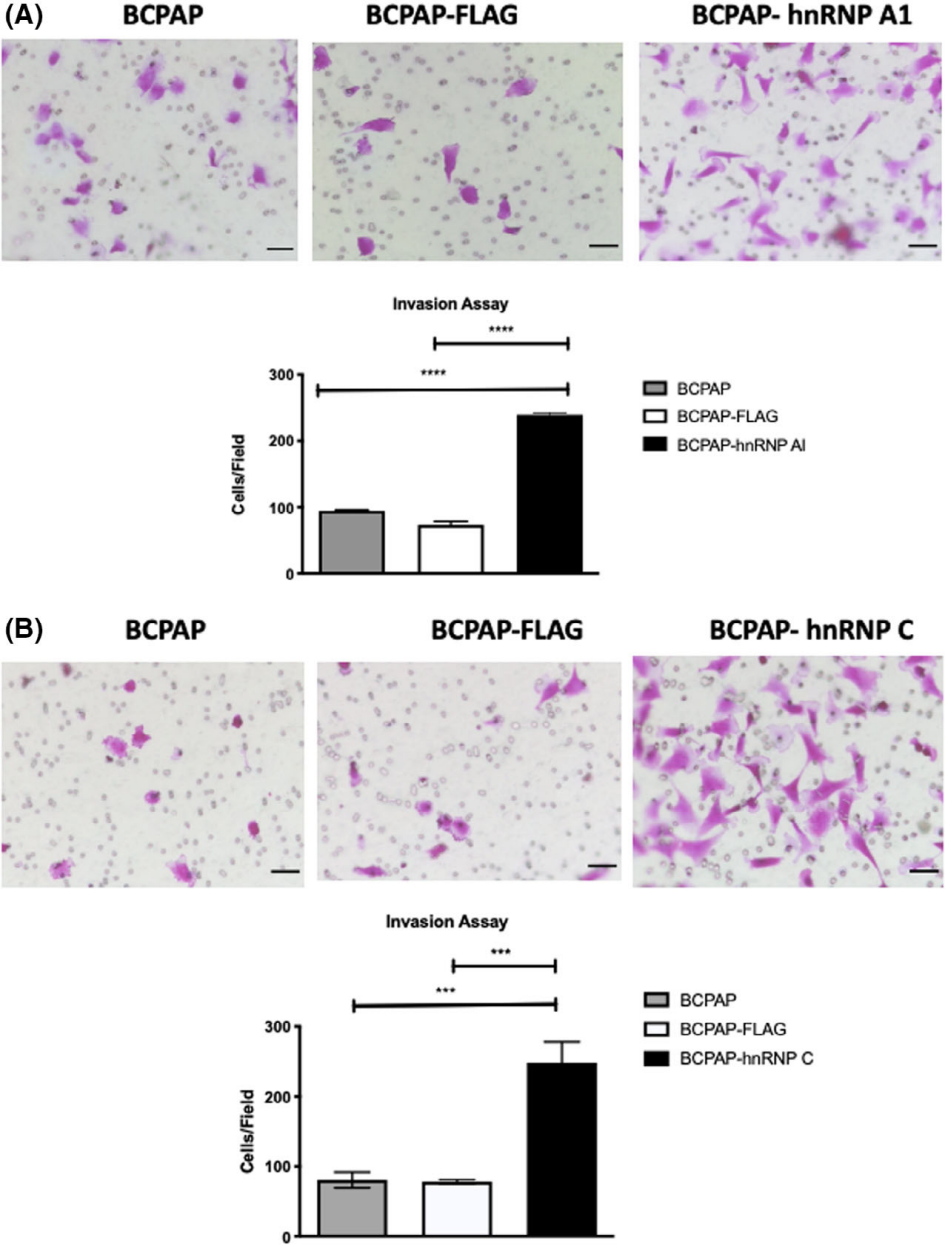
Transwell assays to evaluate cell invasion. Assays were performed on (A) BCPAP‐hnRNP A1 and (B) BCPAP‐hnRNP C cells. BCPAP (untransfected) and BCPAP‐FLAG cells were used as controls. Assays were performed for 6 h. Representative images of the cells traversing the transwell are shown for BCPAP, BCPAP‐FLAG and (A) BCPAP‐hnRNP A1 or (B) BCPAP‐hnRNP C, and the quantification of approximately 10 fields is shown on the graph below (gray bars for BCPAP, white bars for BCPAP‐FLAG and black bars for overexpressing cells). The *y*‐axis indicates the average number of cells per field. Error bars represent standard deviations calculated from 10 fields of at least three different biological samples. Group comparisons were performed using two‐way ANOVA with post‐test Tukey analysis and Student's *t*‐test. ***P < 0.0005; ****P < 0.0001. Scale bars represent 50 µm.

The differences in cell growth upon hnRNP A1 and hnRNP C overexpression indicated these proteins could affect tumor cell biology. We next addressed cell migration and invasion in these cells, two crucial features of tumoral cells. We performed assays using transwell filters to understand whether BCPAP‐hnRNP A1 and BCPAP‐hnRNP C cells have differential migratory and invasive capacities. The observed results indicated that overexpression of hnRNP A1 in BCPAP cells increased these cells' individual migratory and invasive ability compared with the control (Figs 4A and 5A). On the other hand, overexpression of hnRNP C did not significantly modify the migratory capacity of cells since we identified a similar number of cells recovered in BCPAP‐hnRNP C and BCPAP‐FLAG (Fig. 4B). However, cells overexpressing hnRNP C showed increased invasive capacity, indicating the presence of a critical tumoral characteristic (Fig. 5B).

Taken together, our results indicate that increased expression of hnRNP A1 and hnRNP C stimulate expression of miRNAs from the *miR‐17‐92* cluster, especially *miR‐17, miR‐18a* and *miR‐19*, and also lead to increased cell growth and an invasive phenotype. Both hnRNP A1 and hnRNP C are important regulators of intron processing and hnRNP A1 have been associated with *miR‐18a* processing [33]. Modulation of hnRNP A1 and hnRNP C expression can control the development of tumoral features. In addition, the use of antimiRNAs against miRNAs of this cluster might be important to control phenotypic changes commonly observed in tumor cells.

## Discussion

We previously hypothesized that increased hnRNP A1 and hnRNP C concentrations in spliceosomes assembled upon introns containing *miR‐18a* and *miR‐19a* could indicate that these proteins are important splicing regulators for this intron. Thus, if this intron were retained on the mature RNA, we would expect increased levels of these miRNAs. This could lead to cellular modifications that occur during cancer development [20]. In this work, we provide two sets of evidence for the control of expression of these miRNAs by hnRNP proteins; first, hnRNP A1 and hnRNP C specifically bind to the 5′‐end of this pre‐miRNA, and lead to increased levels of miRNAs. Second, we observed that overexpression of these proteins and miRNAs are related to the development of tumorigenic features in thyroid cancer cells.

We first observed that hnRNP A1 and hnRNP C proteins associate to the 5′‐end of the *miR‐17‐92* cluster, causing up‐ or down‐regulation of the miRNAs. HnRNP A1 associates with *miR‐17* and *miR‐18a*, and hnRNP C is strongly associated with *miR‐17, miR‐18a* and *miR‐19a*. Additionally, hnRNP A1 does not associate to *miR‐92*. The presence of binding sites for these proteins on the 5′‐end of this cluster might explain their affinity for miRNAs transcribed from this region, which can lead to increased levels of individual miRNAs. Also, the secondary structure of the pre‐miRNA molecule would lead to a spatial conformation that could facilitate its interaction with regulatory proteins. Previous work suggested that *miR‐92a* is internalized in the secondary structure of the cluster, while other miRNAs would be more exposed and thus could mediate more interactions [13]. The binding of hnRNP A1 to the exposed regions of this cluster might directly affect this intron's splicing efficiency and impact the maturation of the miRNAs, increasing the expression of *miR‐19a* and *miR‐92a*. As for hnRNP C, association to the *miR‐17‐19a* region facilitated *miR‐92a* maturation, but did not affect the expression of other miRNAs. The presence of hnRNPs A/B and hnRNPs F/H in the intron has already been shown to stimulate pre‐mRNA splicing [45]. Similarly, hnRNP A2/B1 has been associated with the miRNAs processing machinery, suggesting the presence of these proteins would also stimulate the microprocessor [46]. Additionally, other spliceosome factors, such as SF3B1 and ISY1, have been shown to be essential for the processing of this pri‐miRNA, reinforcing the role of splicing factors for its biogenesis [47].

Importantly, our results also showed that overexpression of these proteins directly affected the cellular phenotype. Cells overexpressing hnRNP A1 precipitate *miR‐17* and *miR‐18*, and overexpression of hnRNP C led to precipitation of *miR‐17, miR‐18a,* and *miR‐19a*. A higher growth rate is also observed compared with control cells in both cases. Overexpression of hnRNP A1 in another pappilary thyroid cancer cell line, TPC‐1, also enhanced cell growth (data not shown). We interpreted that overexpression of these proteins leads to increased levels of miRNAs from this cluster, stimulating cell growth. These miRNAs are associated with the regulation of pathways related to cell proliferation control. *In silico* analysis performed with target sequences for *miR‐17‐92* cluster showed a high number of targets in genes involved with proliferation, migration, and invasion processes (Fig. S3). *MiR‐19a* is considered a key oncogenic component of the *miR‐17‐92* cluster and is related to several aspects of lymphomas, osteosarcoma, hepatocellular carcinoma, and gastric cancer [18]. Previous studies have demonstrated that *miR‐19a* targets PTEN [48]. PTEN acts by negatively regulating the PI3K‐AKT‐mTOR signaling pathway. In hepatocellular carcinoma*, miR‐19a* downregulates PTEN, and consequently, these cells have increased cell growth rates as AKT is a major player in cell proliferation via mTOR signaling [49, 50]. The transfection of *miR‐19a* inhibitor (antimiR‐19a) in BCPAP cells overexpressing hnRNP A1 and hnRNP C reduced the cell growth rate. This result indicates the phenotype observed might be associated with unbalanced levels of *miR‐19a* through hnRNP A1 and hnRNP C regulation.

Upregulation of *miR‐19a* and *miR‐92a* might also be an important feature for lymphoma progression, affecting cellular phenotype [51]. *MiR‐18a* regulates the PI3K‐AKT‐mTOR signaling by targeting SMG1, an antagonist of mTOR, downregulating SMG1 and consequently increasing PI3K‐AKT‐mTOR signaling. Several studies reported that these miRNAs are also related to increased cell migration and invasion in many cancers. For instance, in colorectal cancer, *miR‐19a* targets TIA1, an important tumor suppressor, leading to cell proliferation and increased migration [52]. Furthermore, *miR‐17*, *miR‐18,* and *miR‐19a* are potential biomarkers for the prognosis of colorectal cancer, nasopharyngeal carcinoma, and osteosarcoma, respectively, as these miRNAs were reported to be often upregulated in these cancers [53, 54]. Additionally, transcription of this cluster is stimulated by c‐MYC, and E2F1 transcription factor is one of the targets of these miRNAs [55, 56].

Previous studies also have shown the importance of hnRNP proteins in mediating the epithelial‐mesenchymal transition (EMT) through phosphatidylinositol 3‐kinase/protein kinase B (PI3K/AKT) in breast and gastric cancers [57, 58]. During EMT, tumor cells acquire migration and invasion capabilities, and then become mesenchymal stem cells [59]. These features are important for tumor progression, as they allow tumor cells to colonize other regions. Indeed, the study of cell migration and invasion is of particular interest, as cancer patients' leading cause of death is related to metastatic progression [60]. We observed increased invasive capacity in cells overexpressing hnRNP A1 and hnRNP C proteins, indicating these proteins might also be important modulators of this process.

hnRNP A1 overexpressing cells also have increased migratory capacity, which might also be related to the upregulation of *miR‐17‐92* miRNAs. Overexpression of *miR‐17* and *miR‐19a* has been linked to increased migratory and invasive rates, acting through the regulation of proteins that participate in processes related to EMT in breast, lung, and colon cancer [43, 61]. Our data suggest increased expression of hnRNP A1 and hnRNP C are responsible for triggering essential tumorigenic processes mainly modulated by alterations in miRNA expression levels. Future studies should explore the roles of hnRNP A1 and hnRNP C on miRNA processing and biogenesis rates in different cell lines. Additionally, further analysis exploring the expression of these proteins in clinical samples and the use of specific molecules targeting the miRNAs of this cluster could greatly improve diagnosis and cancer treatment. A deeper understanding of the roles of hnRNPs on miRNA biosynthesis and processing is of great interest, as this may enable a better understanding of the tumorigenic mechanisms associated with both the regulation promoted by hnRNPs at co‐transcriptional levels and their roles in cancer progression to provide better therapeutic strategies.

## Conclusions

In this paper, we described an important aspect of modulation of thyroid cancer cell malignant phenotype by hnRNP A1‐ and hnRNP C‐dependent regulation of *miR‐17‐92* cluster. The results support our initial hypothesis that hnRNP A1 protein is regulating not only *miR‐18a* expression but also the biogenesis of other members of the *miR‐17‐92* cluster, such as *miR‐19a*. Besides, we observed that overexpression of hnRNP A1 and hnRNP C increased *miR‐17‐92* levels and enhanced pro‐tumorigenic characteristics in BCPAP cells, inducing proliferation, migration, and invasion [57]. Thus, our results extend the molecular mechanisms involving these hnRNPs in the regulation of miRNA processing, especially concerning the *miR‐17‐92* cluster.

## Conflict of interest

The authors declare no conflict of interest.

## Author contributions

PPC, CSF, ETK, and MGPS conceived and designed the project; MGPS, HYN, and GHGS acquired the data; PPC, MGPS, HYN, and GHGS analyzed and interpreted the data; MGPS, CSF, and PPC wrote the paper.

## Supporting information


**Fig. S1.** Basal expression of hnRNP A1 and hnRNP C in thyroid cancer cell lines. Heat color graph from gray to dark blue indicates expression level of the proteins, according to the FPKM (fragments per kilobase) unit. Gray, expression between 0.5 and 10 FPKM; light blue and blue, expression between 10 to 1000 FPKM; dark blue, expression higher than 1000 FPKM (Data from “Expression Atlas and Cancer Cell Line Encyclopedia”; https://www.ebi.ac.uk/gxa/home).
**Fig. S2.** Confirmation of hnRNP A1 and hnRNP C overexpression. BCPAP cells were transfected with plasmids (A) pFLAG‐hnRNP A1 and (B) pFLAG‐hnRNP C (black bars). The control group expressed only the FLAG epitope (white bars). To calculate the change in expression (fold change), normalization with β‐actin amplification was performed. Error bars represent standard deviations calculated from three independent experiments. Group comparisons were performed using two‐way ANOVA with post‐test Tukey analysis and Student's *t*‐test. **P < 0.005.
**Fig. S3.** In silico analysis with the predicted number of targets for miR‐17‐92 miRNAs in thyroid cancer cell lines. X‐axis shows the cell lines and Y‐axis represents the number of predicted targets for *miR‐17‐3p* (blue), *miR‐17‐5p* (orange), *miR‐19a‐3p* (grey) and *miR‐19b‐3p* (yellow). Source: miRDB [37].
**Fig. S4.** Cell viability assay. The viability index of cells (y‐axis) was calculated by counting cells using the Trypan blue exclusion method after 6h. The experiments were performed for (A) BCPAP‐hnRNP A1 and (B) BCPAP‐hnRNP C cells, along with the respective controls (untransfected BCPAP and BCPAP carrying the FLAG epitope). The percentages of viable (white) and dead (black) cells were quantified using the Countess II FL Automated Cell Counter. The percentage values are complementary, and each bar represents an absolute value of 100% of cells (viable + dead cells).

## Data Availability

The data that support the findings of this study are available from the corresponding author (coltri@usp.br) upon request.
