# Supplementary material for: hnRNP A1 and hnRNP C associate with miR‐17 and miR‐18 in thyroid cancer cells
Source: FEBS Open Bio. 2022 Apr 24;12(6):1253–64. doi: 10.1002/2211-5463.13409 (PMC9157402; doi:10.1002/2211-5463.13409)
Supplement: Supplementary file 1 — Fig. S1. Basal expression of hnRNP A1 and hnRNP C in thyroid cancer cell lines. Heat color graph from gray to dark blue indicates expression level of the proteins, according to the FPKM (fragments per kilobase) unit. Gray, expression between 0.5 and 10 FPKM; light blue and blue, expression between 10 to 1000 FPKM; dark blue, expression higher than 1000 FPKM (Data from “Expression Atlas and Cancer Cell Line Encyclopedia”; https://www.ebi.ac.uk/gxa/home). Fig. S2. Confirmation of hnRNP A1 and hnRNP C overexpression. BCPAP cells were transfected with plasmids (A) pFLAG‐hnRNP A1 and (B) pFLAG‐hnRNP C (black bars). The control group expressed only the FLAG epitope (white bars). To calculate the change in expression (fold change), normalization with β‐actin amplification was performed. Error bars represent standard deviations calculated from three independent experiments. Group comparisons were performed using two‐way ANOVA with post‐test Tukey analysis and Student's t‐test. **P < 0.005. Fig. S3. In silico analysis with the predicted number of targets for miR‐17‐92 miRNAs in thyroid cancer cell lines. X‐axis shows the cell lines and Y‐axis represents the number of predicted targets for miR‐17‐3p (blue), miR‐17‐5p (orange), miR‐19a‐3p (grey) and miR‐19b‐3p (yellow). Source: miRDB [37]. Fig. S4. Cell viability assay. The viability index of cells (y‐axis) was calculated by counting cells using the Trypan blue exclusion method after 6h. The experiments were performed for (A) BCPAP‐hnRNP A1 and (B) BCPAP‐hnRNP C cells, along with the respective controls (untransfected BCPAP and BCPAP carrying the FLAG epitope). The percentages of viable (white) and dead (black) cells were quantified using the Countess II FL Automated Cell Counter. The percentage values are complementary, and each bar represents an absolute value of 100% of cells (viable + dead cells). [file FEB4-12-1253-s001.pdf]

Supplementary Figure 1

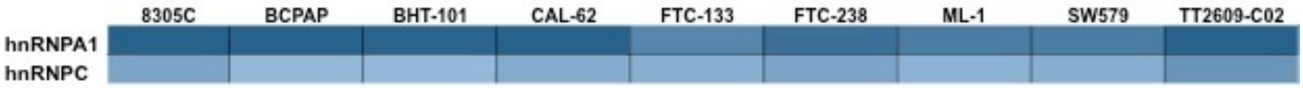

Supplementary Figure 1. **Basal expression of hnRNP A1 and hnRNP C in thyroid cancer cell lines.** Heat color graph from gray to dark blue indicates expression level of the proteins, according to the FPKM (fragments per kilobase) unit. Grey, expression between 0.5 and 10 FPKM; light blue and blue, expression between 10 to 1000 FPKM; dark blue, expression higher than 1000 FPKM (Data from “Expression Atlas and Cancer Cell Line Encyclopedia”; https://www.ebi.ac.uk/gxa/home).

Supplementary Figure 2

A

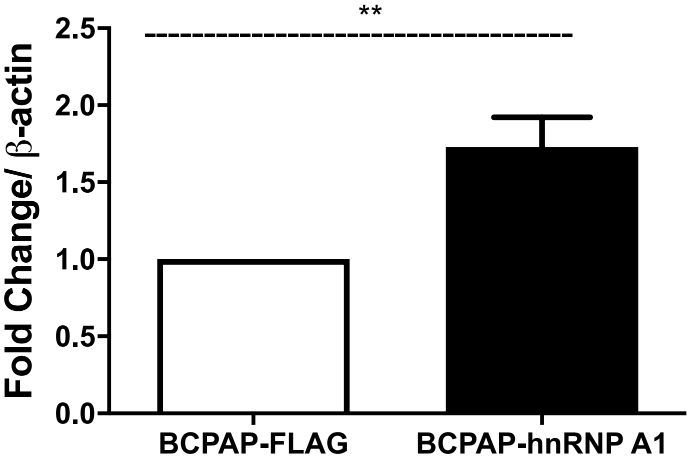

B

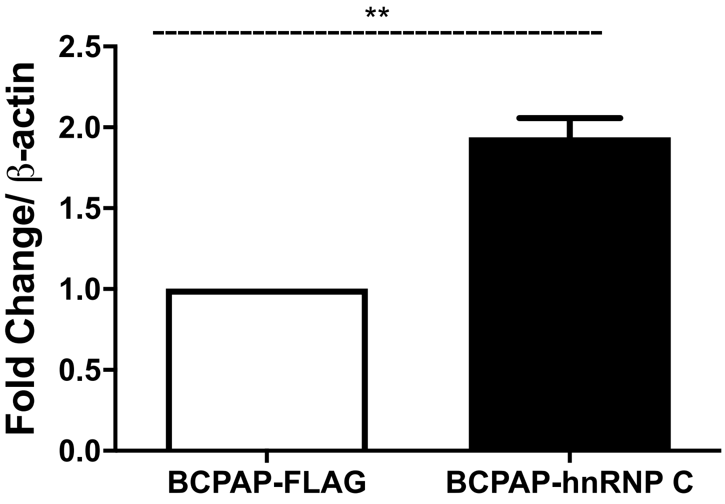

Supplementary Figure 2. **Confirmation of hnRNP A1 and hnRNP C overexpression**. BCPAP cells were transfected with plasmids (A) pFLAG-hnRNP A1 and (B) pFLAG-hnRNP C (black bars). The control group expressed only the FLAG epitope (white bars). To calculate the change in expression (fold change), normalization with β–actin amplification was performed. Error bars represent standard deviations calculated from three independent experiments. Group comparisons were performed using two-way ANOVA with post-test Tukey analysis and Student's t-test. ** P <0.005.

Supplementary Figure 3

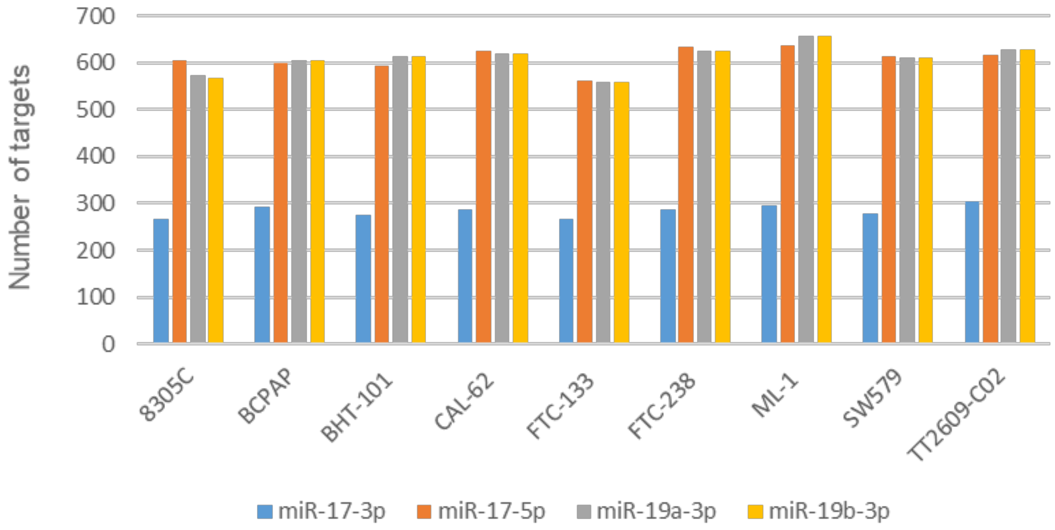

Supplementary Figure 3. **In silico analysis with the predicted number of targets for miR-17-92 miRNAs in thyroid cancer cell lines**. X- axis shows the cell lines and Y-axis represents the number of predicted targets for *miR-17-3p* (blue), *miR-17-5p* (orange), *miR-19a-3p* (grey) and *miR-19b-3p* (yellow). Source: miRDB [37].

Supplementary Figure 4

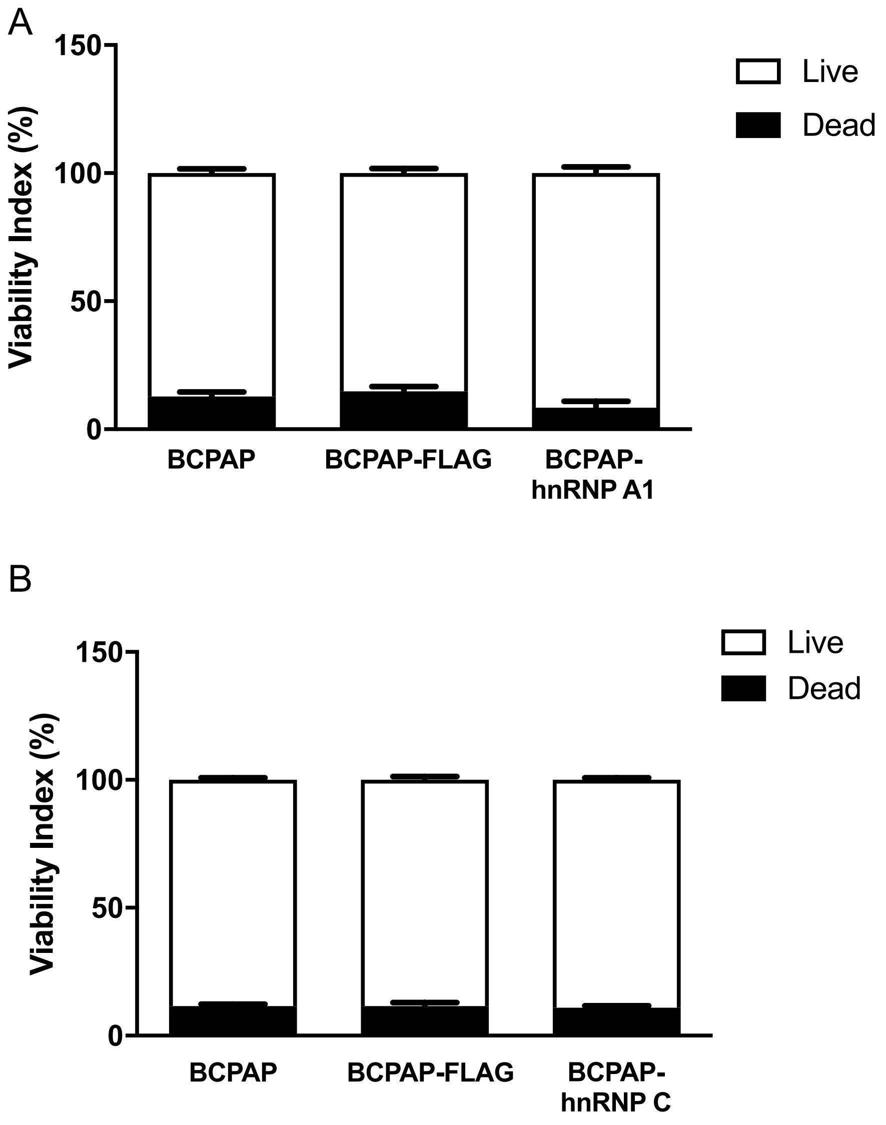

Supplementary Figure 4. **Cell viability assay**. The viability index of cells (y-axis) was calculated by counting cells using the Trypan blue exclusion method after 6h. The experiments were performed for (A) BCPAP-hnRNP A1 and (B) BCPAP-hnRNP C cells, along with the respective controls (BCPAP non-transfected and BCPAP carrying the FLAG epitope). The percentages of viable (white) and dead (black) cells were quantified using the Countess II FL Automated Cell Counters (ThermoFisher). The percentage values are complementary, and each bar represents an absolute value of 100% of cells (viable + dead cells).
